# 

**Published:** 1894-02-17

**Authors:** 


**Notice to Advertisers and Subscribers.**

All Advertisements, Orders for Copies of The Hospital, and Business Communications should be addressed to The Publisher, *not to the Editor*, The Hospital, 428, Strand, W.C.

The Cost of Subscription, post free, is as follows:—Per week, 2½d.; per quarter, 2s. 8d.; per half-year, 5s. 3d.; per annum, 10s. 6d. Foreign:—Per Annum, 12s. 8d.; payable, in all cases, in advance.

**OUR NEW OFFICES.**

428, Strand, W.C., will henceforth be the Office of this Journal.

**Notices of Births, Deaths, and Marriages** are inserted in this column at a charge of 2s. 6d. for an announcement not exceeding four lines.

**NOTICE TO CORRESPONDENTS.**

All MS., Letters, Books for Review, and other matters intended for the Editor should be addressed The Editor, The Lodge, Porchester Square, London, W.

The Editor cannot undertake to return rejected MS., even when accompanied by stamped directed envelope.

**"Burdett's Hospital Annual," 1893.**

Fourth year of publication. 700 pp. Boards, gilt lettering. A most complete guide to British, Colonial, Indian, and American Hospitals, Dispensaries, Nursing and Convalescent Institutions, and Asylums. Price 5s. Published by The Scientific Press (Limited), 428, Strand, W.C.

**NOTICE.—The Index for Vol. XIV. (ending Sept. 30th, 1893) is on sale at the Office of this Journal, price 2½d., post free.**

### Scraps and Gleanings.

The Grimsby Working Men's Hospital Committee have collected £275 towards the Princess May Children's Ward Scheme.

The managers of the Hastings' Dispensary are to be congratulated on the flourishing condition of their finances for the year.

The order of St. Maurice and St. Lazarus has been conferred upon Sir James Lister, M. Pasteur, and Professor Vischow by the King of Italy.

Sir George Elvey has bequeathed £300 each to the Hospital of St. Cross, Rugby, and the Sussex County Hospital for Sick Children at Brighton.

Mr. Algernon Peckover has bequeathed £1,000 to the North Cambridgeshire Cottage Hospital, and not Sir George Elvey, as was erroneously stated.

Miss Ursula Baring has sent a donation of £1,000 to the East London Hospital for Children and Dispensary for Women, Shadwell, for the endowment of a cot.

The medical and surgical report of the County Antrim Infirmary have lately been published. The number of patients admitted to the wards during the year was 328.

A falling off in subscriptions is reported for the Chorlton-upon-Medlock Dispensary. An increase of £4 was reported at the annual meeting on the sale of recommends.

The Bromyard Rural Sanitary Authority have, in consequence of the serious outbreak of small-pox in that district last year, decided to erect a hospital for infectious diseases.

The annual meeting of the Children's Convalescent Home at Meanwood took place recently. New subscribers are much needed, as a deficit was reported to exist on the year's working of the Home.

The annual meeting of the friends of the Manchester Hospital Work Society was recently held. In presenting their fifth report the committee record the continued and satisfactory progress of the society.

The annual meeting of the Manchester Royal Eye Hospital has just taken place. 18,901 patients have received treatment during the year. The balance of income was in excess of expenditure to the amount of £500.

The committee of the Reading Dispensary report that 2,750 new provident members have been admitted. The present estimated membership now numbers 16,266, and the payments by the provident members amount to £2,350.

The total expenditure on the twelve months under review at the annual meeting of the West Cornwall Dispensary and Infirmary was £700, which has left a deficiency of £55 on the working expenses of the institution.

An urgent appeal for support is being made by the directors of the Liverpool Infirmary for Children. A debit balance of £1,000 existed on the accounts last year, but owing to increased support this balance is now reduced to £310.

The annual meeting of subscribers of the Ophthalmic Hospital, Worcester, was recently held, at which it was stated that the total income of the year from every source (excluding legacies) had been £242, whilst the expenditure had been £455.

At the annual meeting of the Turner Memorial Hospital, Keith (N.B.) the treasurer submitted a statement of accounts which showed that the revenue for the year, including a balance in hand, amounted to £312, while the expenditure was only £286.

The twenty-second annual report of the Rotherham Hospital and Dispensary, stated that 82,494 patients had received treatment since the foundation of the institution. In the matter of receipts, the income has steadily increased from year to year.

At a recent meeting at Accrington held in favour of the support towards the cottage hospital for that locality, it was announced that the Mayor had received from two gentlemen members of one family promises of £100 and £50 respectively towards this project.

The annual meeting of subscribers to the Yarmouth Children's Convalescent Home has just taken place. The report states that the financial condition of the home is satisfactory, this being mainly due to the fact that legacies to the amount of £110 have been received.

The governors of St. Bartholomew's Hospital have in contemplation the proposal to purchase some of the land which will be available for building purposes after the boys at Christ's Hospital have found a home in the country. Some extension to the existing building has long been felt to be needed.

A successful meeting was held at Belfast last week to start the movement in connection with raising funds for the new hospital for that town. The money required for this purpose will be at least £60,000, of which £35,000 will go for the purchase of the premises of the Belfast Charitable Society.

In the sixty-third annual report of the Brighton Hospital for Women the treasurer referred to the fact that the new year was commenced with a debt of £142. The money required for the urgent and necessary work of the Building Fund Committee was calculated at £800, only £180 towards which had been so far contributed.

In the twenty-third annual report of the Children's Hospital, Birmingham, it was stated that the number of patients treated during the twelve months was 15,525. The accounts for the year were not reported as satisfactory, the deficiency of income amounting to upwards of £320. Donations towards this hospital have not been so low in any year since 1887.

It is to be hoped that the reporter at a meeting of a northern hospital was responsible for the printed account of the chairman's remark on that occasion. He is put down as having said that surgical cases were expensive because they needed protracted operation! Such a prospect for surgical patients is surely enough to frighten them from the institution.

The annual meeting of the Gorleston Cottage Hospital (Yarmouth) took place recently. The governors having determined that the balance shall in future be made up to December 31st, the present report only deals with nine months; during that period 203 cases have been treated at the hospital. It is a matter of much regret that the local annual subscriptions do not show any increase in numbers.

### Medical Vacancies and Appointments.

**APPOINTMENTS.**

R. C. Bartlett, M.R.C.S., L.R.C.P.Lond., appointed House Surgeon to the Dorset County Hospital. G. H. A. C. Berkeley, M.B., B.C.Cantab., appointed Clinical Assistant to the Ear and Throat Department of the Middlesex Hospital. H. D. Bishop, M.R.C.S.Eng., L.R.C.P.Lond., appointed Senior House Surgeon to the Carnarvon Hospital, Kimberley. H. J. L. Bullen, M.R.C.S.Eng., L.S.A., appointed Medical Officer for the Third District of the New Forest Union. L. J. Hobson, M.D.Lond., B.S., F.R.C.S., reappointed Honorary Consulting Physician to the Royal Bath Hospital and Rawson Convalescent Home, Harrogate. P. J. Jackson, M.R.C.S.Eng., appointed Medical Officer to the Southwark Division of the General Post Office. R. L. Jones, M.R.C.S.Eng., &c., appointed Medical Officer to the North Wales Church Training College, Bangor. C. P. O'Connor, M.D., appointed Medical Officer for the Third District of the North Witchford Union. J. W. G. Prince, M.R.C.S., L.R.C.P.Lond., appointed Medical Officer for the Third District of East Grinstead Union. R. H. Shaw, B.A., M.B., B.Ch.Univ. Dub., appointed Acting House Surgeon to Mercers' Hospital, Dublin. J. P. Tildesley, M.R.C.S., L.R.C.P.Lond., appointed Medical Officer of the Willenhall District of the Wolverhampton Union. J. A. Unitt, L.R.C.P.Edin., M.R.C.S.Eng., appointed Medical Officer for the Quorn District of the Barrow Union. W. H. Walter, M.D.Brux., L.R.C.P.Edin., M.R.C.S., appointed Medical Officer for the Second District of the Winslow Union. H. F. Waterhouse, M.D., C.M.Edin., F.R.C.S.Eng., appointed Aural Surgeon to Charing Cross Hospital. G. H. Wickham, M.B., B.C.Cantab., appointed Honorary Medical Officer for Out-patients, Royal Victoria Hospital, Bournemouth. C. G. R. Wood, M.R.C.S.Eng., L.R.C.P.Lond., appointed Honorary Ophthalmic Surgeon to the North of England Children's Sanatorium. P. M. Yearsley, F.R.C.S.Eng., appointed Curator of Museum, Westminster Hospital. Fred. W. Perry, L.R.P.I., L.R.C.S.I., L.M.Dublin, appointed House Surgeon to the Pendleton Branch Dispensary, vice John Valentine, deceased.

**VACANCIES.**

**Resident Medical Officers.**

East London Hospital for Children, Shadwell, E. Pathologist and Registrar. Honorarium £40 per annum. Applications to the Secretary, Thomas Hayes, by February 27th.

County Lunatic Asylum, Sneaton, Nottingham. Assistant Medical Officer, unmarried. Salary £100 per annum, rising £10 annually to £150, board, lodging, washing, and attendance. Applications to the Chairman of the Committee of Visitors by February 27th.

Cumberland Infirmary, Carlisle. Assistant House Surgeon. Salary £40 per annum, with board, lodging, and washing. Appointment for one year. Applications and testimonials to the Secretary by February 21st.

East Suffolk and Ipswich Hospital, Ipswich. House Surgeon, unmarried. Qualified in medicine and surgery. The office is held subject to annual re-election. Salary £80 per annum, with board, lodging, and washing. Applications and testimonials to the Secretary, T. Edgar Mayhew, by February 20th.

Hospital for Women, Soho Square, W. House Physician. Salary £30 for six months, with board, &c. Applications and testimonials to the Secretary, David Cannon, by February 21st.

Hospital for Women and Children, Leeds. House Surgeon for less than twelve months. Salary £75 per annum. Applications to the Secretary of the Faculty.

Oxford Eye Hospital. House Surgeon. Appointment for one year. Salary £50, with board and lodging. Applications to Mr. B. H. Baden-Powell, Honorary Secretary, 29, Banbury Road, Oxford, by February 24th.

Queen Charlotte's Lying-in Hospital, Marylebone, N.W. Resident Medical Officer. Appointment for four months. Salary at the rate of £60 per annum, with board and residence in the hospital. Applications and testimonials to the Secretary, G. Owen Ryan, by February 20th.

Royal Surrey County Hospital, Guildford. House Surgeon. Salary £80 per annum, with board, lodging, and laundry. Applications to the Honorary Secretary by March 10th.

Staffordshire County Infirmary, Stafford. Assistant House Surgeon. No salary, but board, lodging, and washing. Applications to House Surgeon.

Tiverton Infirmary and Dispensary, Tiverton. House Surgeon and Dispenser, registered and unmarried. Salary £100 per annum, with lodgings, attendance, fire, and lights. Applications with testimonials to the Honorary Secretary, Arthur Fisher, Tiverton, Devon, by February 23rd.

**Non-Resident Medical Officers.**

St. Luke's Hospital, London, E.C. Clinical Assistant. Appointment for six months, with board and residence. Applications and testimonials to the Secretary, Percy De Bathe, M.A., by February 19th.

Wynaad Planters' Association. Medical Officer for an Indian planting district. Salary 450 rupees a month. Married man preferred. Applications to J. Williams Hockin, Honorary Secretary, U. P. A. Medical Fund, Vayitiri, Malabar, India.

### BOOKS RECEIVED.

SIMPKIN MARSHALL.

"The Great Pestilence." By Francis A. Gasquet, D.D.

KELLY AND CO.

"Kelly's London Medical Directory, 1894."

SAMPSON LOW, MARSTON, AND CO.

"Rambles in Books." By Charles F. Blackburn.

LONGMANS, GREEN, AND CO.

"A Gentleman of France." By Stanley F. Weyman.

"The Student's Dictionary of Medicine." By Alexander Duane, M.D.

A. AND C. BLACK, LONDON.

"Investigations on Microscopic Foams and on Protoplasm." By O. Butschli. Translated by E. Minchin, B.A.

F. KIRBY, LONDON.

"The Englishwoman's Year-Book, 1894."

GEORGE ALLEN, LONDON.

"The Oxford Museum." By Henry Acland, M.D., and John Ruskin, M.A.

